# Prostate cancer inducing secondary linitis plastica of the rectum: a rare case report and literature review

**DOI:** 10.3389/fonc.2025.1597367

**Published:** 2025-07-23

**Authors:** Dongpo Zhang, Jun Li, Tao Sun, Ling Zhang, Lian Wang, Quan Gan, Xiaoxiao Xing, Yong Zhang, Yue Wang, Daixiang Liao, Junyi Li

**Affiliations:** ^1^ Department of Surgery, Guang’anmen Hospital, China Academy of Chinese Medical Sciences, Beijing, China; ^2^ Department of Anorectal Surgery, Guang’anmen Hospital (Baoding), China Academy of Chinese Medical Sciences, Beijing, China; ^3^ Department of Pathology, Guang’anmen Hospital, China Academy of Chinese Medical Sciences, Beijing, China

**Keywords:** prostate cancer, secondary rectal linitis plastica, rectal adenocarcinoma, PSA, MRI

## Abstract

**Background:**

Prostate cancer, the most prevalent male malignancy in Western countries, seldom presents as secondary rectal linitis plastica (RLP).

**Case presentation:**

We present an 82-year-old man with a 6-month history of altered bowel habits, narrowed stools, and mucous discharge, with absent lower urinary tract symptoms. Serum Prostate Specific Antigen (PSA) was markedly elevated (392 ng/mL). Imaging demonstrated circumferential rectal thickening and a prostatic mass invading the bladder. MRI revealed a “target sign” with associated diffusion restriction. Colonoscopy identified circumferential mucosal protrusions resembling grape-like clusters (Nice Band Imaging (NBI) International Colorectal Endoscopic (NICE) type 3). Deep biopsies confirmed prostatic adenocarcinoma (Gleason score 4 + 3 = 7).

**Diagnosis:**

A multidisciplinary team confirmed the diagnosis of prostate cancer with secondary RLP.

**Treatment:**

Combination therapy (prophylactic colostomy, leuprorelin, and abiraterone) reduced PSA from 392 to 2.16 ng/mL within 8 months.

**Conclusions:**

RLP may mimic various gastrointestinal disorders clinically. Clinicians should consider RLP in elderly men presenting with gastrointestinal symptoms. Definitive diagnosis requires the integration of multi-modality imaging, endoscopy, and histopathological biopsy.

## Highlights

- RLP may mimic different diseases in presentation.
- Clinicians should suspect RLP in elderly men with gastrointestinal symptoms. Collaboration across specialties may avoid misdiagnosis.
- The combination of different imaging modalities, endoscopy, and tissue biopsy leads to definitive diagnosis.

## Background

Prostate cancer is the most prevalent malignancy and the second leading cause of cancer-related mortality in Western men (1). Bone metastases constitute the predominant metastatic pattern, followed by lymphatic, pulmonary, and hepatic involvement. Clinically, prostate cancer primarily presents with lower urinary tract symptoms: 1) irritative symptoms (urinary frequency, urgency, nocturia, and urge incontinence), 2) obstructive symptoms (hesitancy, weak stream, intermittency, and retention), and 3) local invasion symptoms (testicular pain, painful ejaculation, hematuria, renal insufficiency, and hematospermia). Denonvilliers’ fascia forms the primary anatomical barrier between the rectum and prostate, critically containing tumor spread. The disruption of this fascial layer enables direct prostate cancer invasion into the rectum (2).

Secondary rectal linitis plastica (RLP) represents a rare metastatic manifestation. Its pathogenesis involves direct tumor extension or lymphatic dissemination from prostate cancer to the rectal wall. Conventional rectal invasion by prostate cancer typically demonstrates localized anterior wall involvement on imaging (3), whereas RLP features diffuse circumferential fibrosis and mural thickening throughout the rectum. Histopathological examination reveals diffuse tumor cell infiltration through the submucosal and muscularis propria layers, with prominent desmoplastic stromal reaction (4). Clinically, RLP manifests predominantly with gastrointestinal symptoms including defecatory dysfunction (80%), rectal pain (60%), and weight loss (40%), mimicking primary rectal adenocarcinoma. This report illustrates a rare case of prostate cancer-induced secondary rectal linitis plastica.

## Case presentation

**Figure 1:**
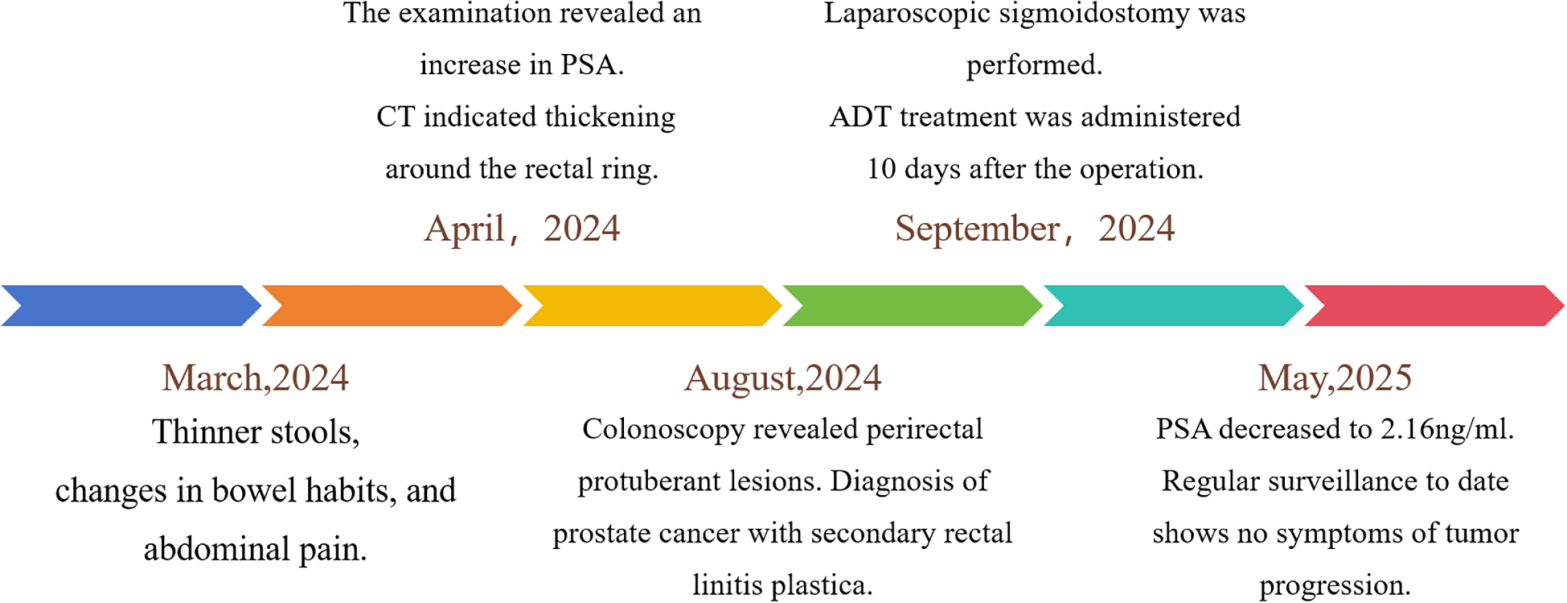
Clinical timeline: symptom onset, diagnosis, and treatment.

An 82-year-old man presented to our department with a 6-month history of altered bowel habits. **Figure 1** illustrates the diagnostic and therapeutic timeline. Six months prior, he developed narrowed stools, increased defecation frequency (6–10 daily), defecation-related abdominal pain, persistent post-evacuation urgency, and mucoid stools. Notably, he denied hematochezia, dysuria, night sweats, or weight loss. Initial tumor markers revealed the following: total PSA 392 ng/mL (ref: <4), free PSA 49.7 ng/mL, and free prostate-specific antigen / total prostate-specific antigen (fPSA/tPSA) ratio of 0.13. Contrast-enhanced abdominal computed tomography (CT) demonstrated the following: circumferential wall thickening (max 2.0 cm) at 4 cm from the anal verge and serosal penetration with ill-defined prostate borders in the rectum, enlargement (38 × 53 mm) with extracapsular bladder invasion and seminal vesicle angle obliteration in the prostate, and complication of bilateral hydroureteronephrosis (left, 2.1 cm; right, 1.8 cm). In August 2024, the patient was referred to our surgical department.

### Physical examination

Digital rectal examination revealed a fixed stenotic mass at 5 cm from the anal verge, preventing further advancement. The prostate was markedly enlarged and indurated, and blood was noted on digital rectal examination.

#### Auxiliary examinations

**Figure 2:**
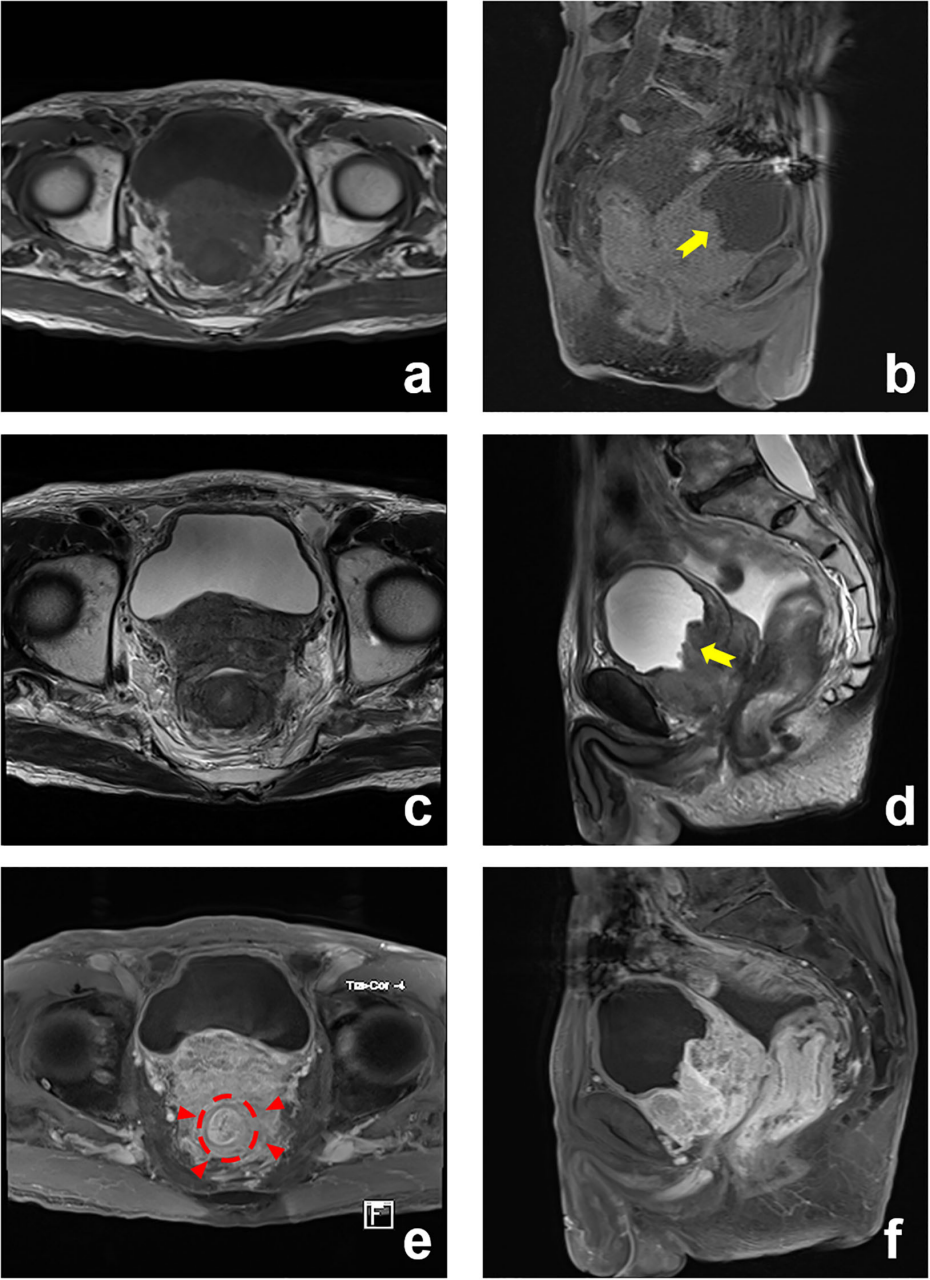
Prostate MRI: enlarged prostate with transition zone enlargement. Posterior extension: encasement of the rectum (“target sign”) (red arrowed). Anterior extension: invasion of the bladder (yellow arrowed). **(a)** T1-Weighted (T1W), axial; **(b)** T1-Weighted (T1W), sagittal; **(c)** T2-Weighted (T2W), axial; **(d)** T2-Weighted (T2W), sagittal; **(e)** T1-Weighted (T1W) C+, axial; **(f)** T1-Weighted (T1W) C+, sagittal.

**Figure 3:**
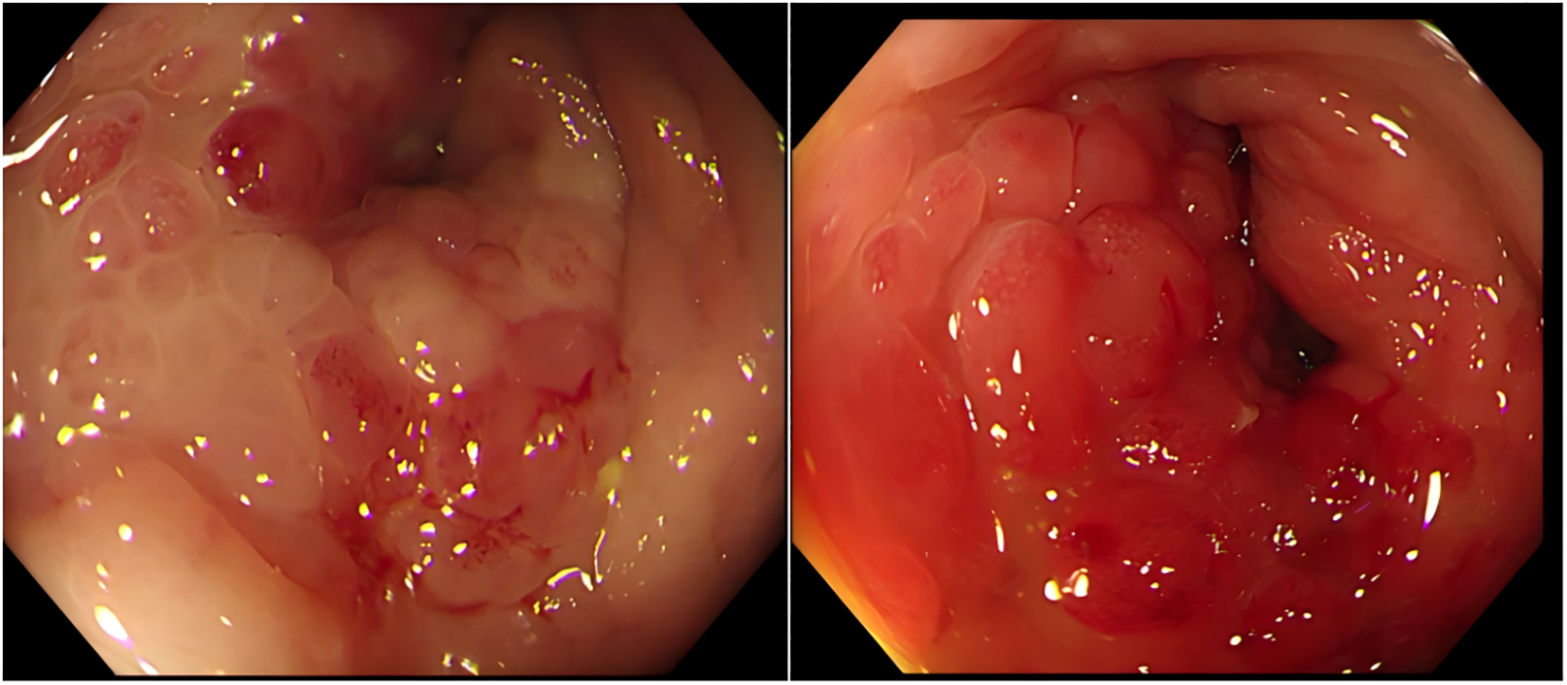
Colonoscopic findings. Colonoscopy demonstrated rectal stenosis with circumferential mucosal protrusions resembling grape-like clusters, characterized by friability and contact bleeding.

**Figure 4:**
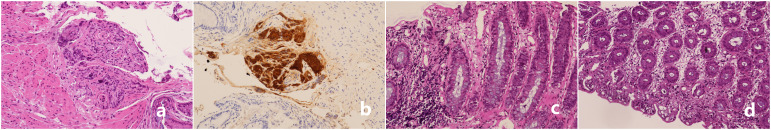
Histopathological findings. **(a, b)** Tumor cells infiltrating the muscularis mucosae and submucosa, displaying small acinar structures with crowded, disorganized glands and dysplastic acini, consistent with prostatic acinar adenocarcinoma (Gleason score 4 + 3 = 7) (H&E, ×200). **(c, d)** Adjacent mucosa shows superficial erosion, stromal edema, fibrous hyperplasia, and dense lymphocytic infiltration (H&E, ×100).

Subsequent contrast-enhanced pelvic MRI revealed a rectal tumor with infiltrative carcinoma and concentric full-thickness rectal wall involvement. Posterior extension demonstrated circumferential encasement of the rectum exhibiting “target sign” (**Figure 2**). Colonoscopy revealed a circumferential elevated lesion spanning 4–7 cm from the anal verge in the rectum (**Figure 3**). The lesion exhibited circumferential mucosal protrusions resembling grape-like clusters. Narrow-band imaging chromoendoscopy revealed a NICE type 3 pattern, indicating deep submucosal invasion. The endoscope could not be fully advanced through the stenotic segment (**Figure 3**). Through colonoscopic biopsy pathology, histopathological examination of colonoscopic biopsies revealed infiltrative atypical glands extending beyond the muscularis mucosae, with prominent nucleoli. Immunohistochemical analysis demonstrated positive PSA, positive prostate-specific membrane antigen (PSMA), negative Carcinoembryonic Antigen (CEA), and a Ki-67 labeling index of 10%–20%. The findings were consistent with metastatic prostatic acinar adenocarcinoma (Gleason score 4 + 3 = 7) (**Figure 4**).

#### Diagnosis

Based on elevated PSA levels, imaging features, and colonoscopic biopsy histopathology, a multidisciplinary team (MDT) comprising specialists in urology, surgical oncology, pathology, and radiology convened for diagnostic assessment. The patient was definitively diagnosed with prostate cancer accompanied by secondary RLP.

#### Treatment and prognosis

Given locally advanced disease with bladder invasion and rectal lymphatic permeation, definitive surgical management necessitated total pelvic exenteration. The 72-year-old male patient presented with a low body mass index (BMI 16.2 kg/m^2^) and severe malnutrition. His nutritional status was poor. The patient’s current frail physical condition rendered him unable to tolerate such a major procedure. Consequently, emergency management focused on relieving malignant bowel obstruction. Definitive treatment (either radical surgery or localized radiotherapy) was deferred until nutritional status improved. The MDT consensus recommended diverting sigmoid colostomy for palliation of obstruction. According to the clinical practice guidelines for prostate cancer (5), androgen deprivation therapy (ADT) with leuprorelin and abiraterone was administered to the patient. Laparoscopic sigmoid loop colostomy was performed in September 2024. ADT commenced on postoperative day 10.

#### Follow-up

Serial PSA monitoring demonstrated progressive decline: 164 (1 month), 53 (3 months), 5.89 (6 months), and 2.16 ng/mL (8 months) post-ADT. The patient elected to forgo surveillance imaging, such as bone scintigraphy and PSMA-positron emission tomography (PET)/CT. The initial ADT regimen (leuprorelin with abiraterone acetate) was maintained. Ongoing surveillance revealed no clinical evidence of disease progression.

## Discussion

This case exhibited exclusively gastrointestinal manifestations: malignant bowel obstruction, decreased stool caliber, increased bowel movement frequency, and incomplete defecation, with absent lower urinary tract symptoms. Putative metastatic pathways to the rectum included the following: a) direct invasion through Denonvilliers’ fascia, b) lymphatic permeation, c) retrograde venous spread, and d) iatrogenic implantation post-transrectal biopsy (4). Tumor infiltration induced desmoplastic stromal reaction in the rectal submucosa, causing muscularis propria rigidity and contracture that culminated in luminal stenosis. MRI demonstrated circumferential encasement of the rectum by prostatic cancer, with tumor extension posteriorly involving Denonvilliers’ fascia. This generated concentric layered thickening of the rectal wall, classically termed the “target sign” on imaging (6).

We reviewed similar reported cases in the literature, as shown in **Table 1** (3, 4, 6–8). This comparative analysis of seven published cases revealed that RLP secondary to prostate cancer occurs exclusively in elderly men (ages 57–86), presenting primarily with bowel dysfunction (constipation, incontinence, pain, and bleeding) or weight loss, although one case was asymptomatic. Crucially, half of the cases had no prior cancer diagnosis. Diagnosis relied on MRI, PSMA-PET, and histology. ADT was the main reported treatment. Outcomes varied considerably, ranging from symptomatic improvement to death or progression.

**Table 1:** Clinical features, diagnostics, treatments, and outcomes of similar cases.

Case	Age	Sex	Symptoms	History of past illness	Imaging examinations	Diagnostics	Treatments	Outcomes	Reference
Case 1	66	Male	Constipation, tenesmus, fecal incontinence, loss of weight	No history of malignancy	magnetic resonance imaging (MRI)	Prostate cancer causing secondary RLP	ADT as the sole therapy	Serum PSA decreased, and his bowel habit improved significantly	(7)
Case 2	70	Man	Bowel habit with non-bloody loose stool three times a day, abdominal pain, loss of weight	No history of malignancy	MRI	RLP secondary to prostate carcinoma	ADT (goserelin) in combination with cyproterone	The patient contracted COVID-19 and died approximately 10 months after the diagnosis of RLP	(8)
Case 3	61	Man	Difficulty in defecation with episodic bloody stools	No history of malignancy	68Ga-PSMA-11 PET/CT and PET/MRI	RLP secondary to prostate adenocarcinoma	Unknown	Unknown	(6)
Case 4	86	Man	Diarrhea and occasional rectal bleeding	No history of malignancy	MRI	Prostate cancer invading the rectum	Colonoscopy and ADT	Approximately 3 years after, patient was still alive, but PSA level increased	(3)
Case 5	76	Man	Rectal pain and fecal incontinence	Prostate cancer	MRI	RLP due to prostatic adenocarcinoma	Not available at the time of reporting	Not available at the time of reporting	(4)
Case 6	57	Man	No symptoms, elevated PSA levels during routine follow-up	Prostate cancer	MRI and (PET/CT) PSMA	RLP due to prostatic adenocarcinoma	Not available at the time of reporting	Not available at the time of reporting	(4)
Case 7	80	Man	Refractory constipation	Prostate cancer	MRI	RLP due to prostatic adenocarcinoma	Not available at the time of reporting	Not available at the time of reporting	(4)

RLP, rectal linitis plastica; ADT, androgen deprivation therapy.

Our case of secondary RLP aligns with the core demographic (elderly man) and clinical presentation (bowel dysfunction) seen in published cases. It underscores the critical diagnostic challenges of frequent absence of prior cancer history (like Cases 1–4) and the pitfall of normal mucosa or superficial biopsies necessitating deep sampling. Radiologically, it confirms the centrality of MRI and adds emphasis on the suggestive “target sign” and Diffusion-Weighted Imaging (DWI) restriction. The unique “grape-like” endoscopic morphology provides a valuable descriptive feature. Therapeutically, it demonstrates a robust early response to a contemporary, intensified hormonal regimen (ADT + abiraterone) and combined with proactive surgical management (colostomy) for obstruction. While sharing the underlying pathology, our case enhances the literature by detailing specific diagnostic features (imaging signs, endoscopic morphology, and biopsy strategy), reporting on a novel treatment combination, and documenting a significant early biochemical response.

PET/CT imaging using isotope-labeled PSMA ligands is essential for diagnosis and prognosis in prostate cancer patients. PSMA-PET/CT is superior to conventional imaging (MRI, CT, and bone scan) in primary staging, mainly in the detection of pelvic lymphadenopathy and distant metastases. PSMA-PET/MRI is an emerging modality that combines metabolic information on PSMA receptor expression in prostate tumors derived from PET, with anatomical and functional information derived from magnetic resonance (MR) in one procedure. PSMA-PET/MR is accurate and reliable in the depiction of nodal and osseous metastases compared with PSMA-PET/CT (9, 10). Unfortunately, the patient elected to forgo surveillance imaging.

RLP poses a challenge by closely mimicking primary rectal adenocarcinoma both clinically (rectal bleeding/mass) and endoscopically. However, the biopsy revealed adenocarcinoma with morphology suggestive of prostatic origin. Immunohistochemistry confirmed prostatic lineage (positive for PSA). The Immunohistochemistry (IHC) profile was definitive in distinguishing metastatic prostate cancer from primary rectal cancer. Based on the elevated PSA, radiological findings, and colonoscopy biopsy pathology, a multi-disciplinary team involving specialists from general surgery, urology, oncology, pathology, and radiology held a discussion. The patient was definitively diagnosed with prostate cancer accompanied by secondary RLP.

Diagnosing prostatic adenocarcinoma with rectal invasion poses significant challenges. RLP typically involves submucosal infiltration by prostate cancer cells, often leaving the overlying rectal mucosa intact or only superficially involved. In approximately 30% of RLP cases, the rectal mucosa appears endoscopically normal due to tumor confinement to the submucosal and muscularis propria, often resulting in false-negative initial biopsies and necessitating deep-tissue sampling for definitive diagnosis (8). Superficial biopsies typically demonstrate mucosal erosion, stromal edema, and reactive fibroinflammatory hyperplasia. Such findings frequently delay definitive diagnosis. Deep targeted biopsies sampling the lesion base or submucosal nodules (grape-cluster morphology) are critical for detecting underlying malignancy (11). Initial external institutional biopsies revealed reactive hyperplasia without malignant evidence. Repeat deep biopsies at our center specifically sampled submucosal and muscularis mucosa layers, confirming invasive prostatic adenocarcinoma.

## Conclusion

Secondary RLP in prostate cancer is an aggressive and diagnostically elusive entity. Clinicians should maintain a high index of suspicion in elderly men with rectal wall thickening or suspected rectal tumors, particularly those with a history of prostate cancer. A multimodal diagnostic approach—integrating clinical history, serum PSA, advanced imaging (MRI), and deep tissue sampling—is critical to avoid misdiagnosis and ensure histopathological confirmation.

## Limitations

This case report provides unique educational insights into the diagnostic challenges and management of prostate cancer metastasis to the rectum. As a case report, conclusions may not be generalizable, and more clinical studies are needed.

## Data Availability

The original contributions presented in the study are included in the article/Supplementary Material. Further inquiries can be directed to the corresponding author.
